# Perinatal Palliative Care Birth Planning as Advance Care Planning

**DOI:** 10.3389/fped.2020.00556

**Published:** 2020-09-08

**Authors:** DonnaMaria E. Cortezzo, Kelstan Ellis, Amy Schlegel

**Affiliations:** ^1^Division of Neonatal and Pulmonary Biology, Cincinnati Children's Hospital Medical Center, Cincinnati, OH, United States; ^2^Division of Pain and Palliative Medicine, Cincinnati Children's Hospital Medical Center, Cincinnati, OH, United States; ^3^Department of Pediatrics, University of Cincinnati College of Medicine, Cincinnati, OH, United States; ^4^Department of Anesthesiology, University of Cincinnati College of Medicine, Cincinnati, OH, United States; ^5^Department of Pediatrics, University of Missouri Kansas City, Kansas City, MO, United States; ^6^Section on Palliative Care, Children's Mercy Kansas City, Kansas City, MO, United States; ^7^Division of Neonatology, Nationwide Children's Hospital, Columbus, OH, United States; ^8^Department of Pediatrics, Ohio State University of Medicine, Columbus, OH, United States

**Keywords:** perinatal palliative care, birth plan, advance care planning, neonatal, life-limiting diagnosis

## Abstract

**Purpose of Review:** A significant number of pregnancies are complicated by a fetus with a life-limiting diagnosis. As diagnoses are made earlier in the pregnancy, families experience anticipatory grief and are faced with navigating goals of care for a baby that has yet to be born. With the support of the care team, families can begin to grieve, plan, and make meaningful memories during the duration of the pregnancy, the birth of their baby, and life of the child. Creating a palliative care birth plan, which expands beyond the traditional concept for delivery planning to include prenatal, perinatal, and neonatal care has become an important method for parents to process the diagnosis, for parents to document their wishes, and for members of the care team to communicate with the goal of supporting and enhancing the experience of the family. This articles reviews recent and relevant literature on the importance of birth planning and the role of perinatal palliative care when a life-limiting fetal diagnosis is made.

**Recent Findings:** The process of birth planning is an important component of perinatal palliative care. Through this process, families can express their fears, values, hopes, and wishes. It also offers an opportunity for providers to communicate these wishes for the remainder of the pregnancy, the delivery, birth, and time afterwards. This has been demonstrated to decrease maternal stress and promote family centered care.

**Summary:** Perinatal birth planning is an important component of perinatal palliative care when a fetus has a life-limiting diagnosis. The process of birth planning can be supportive and therapeutic as well as an important communication tool. With multiple practices and designs of perinatal palliative care programs, there are no standard tools even though important components have been identified. Ultimately, the strategies outlined here can be used as advance care planning tools.

## Introduction

Advances in prenatal care and diagnostic technologies have allowed for earlier and more frequent identification of life-limiting fetal diagnoses, leading to increased numbers of families seeking support in decision-making surrounding the pregnancy, delivery, and neonatal care of medically complex newborns (1–3). Perinatal palliative care (PnPC) consultation as a part of family-centered fetal care offers opportunities for exploration of goals of care and planning for value-driven medical care for the remainder of the pregnancy, during delivery, and in the neonatal period (4–8). While practices vary and some PnPC teams investigate a spectrum of options that includes interruption of pregnancy, most PnPC consultation occur once a family decides to continue a pregnancy with a potentially life-limiting fetal diagnosis. PnPC teams ensure that families demonstrate sound medical understanding and prognostic awareness. They aid in exploration of unique value-systems from which families may choose various pathways including expectant pregnancy management, close monitoring of the pregnancy, interventions during labor and delivery, comfort-focused newborn care, or subspecialty neonatal evaluation and medical interventions. PnPC providers advocate for clear communication and support each family in vocalizing goals of care and wishes for their child's life (9).

Creation of a palliative care birth plan is an important method of exploring and documenting requests for the remainder of the pregnancy, labor and delivery, and the subsequent care of a newborn with a life-limiting diagnosis (10–12). The process of palliative care birth planning is both functional and therapeutic for families navigating goals of care discussions. Ultimately a care plan, the culmination of the important birth planning process, is developed to document and communicate the family's wishes with the multidisciplinary medical team. In this way, the palliative care birth plan expands beyond the traditional concept of a birth plan, which communicates preferences for the logistics surrounding delivery, to include prenatal, perinatal, and neonatal care. The PnPC birth plan can give families a sense of control in a challenging time and an opportunity to honor the life of their child. This article reviews recent literature on PnPC birth planning in the setting of a life-limiting fetal diagnosis and provides recommendations on developing documents that can be used as effective communication tools in advance care planning.

## Potentially Life-Limiting Fetal Diagnoses

In the United States alone, there are over 1 million fetal deaths and over 15,000 neonatal deaths each year (13). The majority of these, with up to 3% of pregnancies complicated by a potentially life-limiting fetal diagnosis, are the result of congenital malformations or chromosomal anomalies (14–16). Expanded early genetic screening or diagnostic options and advances in imaging technology have resulted in earlier and more frequent identification of life-limiting fetal diagnoses. A life-limiting condition is one having a low likelihood of long-term survival without severe morbidity that could impact quality of life. Multidisciplinary fetal care centers have expanded to include perinatal palliative care specialists who assist families facing complicated pregnancies in exploring options for care of the fetus and newborn (1, 3, 17). Some fetuses with life-limiting diagnoses will die *in utero*. Others may die during labor and delivery or in the immediate neonatal period. Others still may survive with varying degrees of interventions for hours, days, or more (1, 10, 18). Within these diagnoses are a wide range of pathology with a high lethal potential where individuals are expected to have varying degrees of survival and life spans. Table 1 shows a list of common potentially life-limiting fetal diagnoses that perinatal palliative care teams are commonly consulted for.

**Table 1:** Examples of common potentially life-limiting fetal diagnoses.

**Genetic/Metabolic** • Trisomy 13 • Trisomy 18 • Osteogenesis imperfect (severe phenotypes) • Triploidy • CPT2 deficiency **Cardiac** • Acardia • Inoperable lesions • Lesions with poor prognosis despite palliative surgery options **Central nervous system** • Anencephaly • Alobar holoprosencephaly • Pontocerebellar hypoplasia • Hydrancephaly • Giant encephaloceles • Spinal muscular atrophy **Severe pulmonary hypoplasia** • Bilateral renal agenesis with anhydramnios (Potter's sequence) • Thanatophoric dysplasia and other severe skeletal dysplasias • Short rib polydactyly syndrome • Congential diaphragmatic hernia with low O:E lung volumes • Severe hydrops fetalis **Other** • Severe pentalogy of cantrell • Limb-body wall complex • Severe forms of conjoined twins

With opportunities for multidisciplinary evaluation and increased access to perinatal palliative care, more women, between 20 and 40%, are choosing to continue pregnancies with a fetal life-limiting diagnosis (11, 19). Of those choosing to continue the pregnancy, reports of live births range from 60 to 82% (11, 20–23). When a child survives into the neonatal period, families face many decisions surrounding treatment and approaches to care. Some of these options may alter the disease trajectory but are unable to correct the underlying physiology or significantly alter the prognosis of the condition for the child. Earlier and enhanced fetal diagnosis gives families time to receive multidisciplinary fetal care with expert explanation of the suspected or confirmed diagnosis, support in understanding the prognosis and accepting levels of uncertainty, and value-driven shared decision-making in determining appropriate care or treatment options. With this information and support, families can utilize the ongoing prenatal period to create and share hopes, requests, and goals for the remainder of the pregnancy and their child's care after delivery, enhancing opportunities to parent in the face of a life-limiting diagnosis.

The response to a life-limiting fetal diagnosis is unique to each family and their pregnancy journey. Some families seek comfort-focused medical care for the remainder of the pregnancy and for their child while others seek support in exploring or requesting varying degrees of life-sustaining interventions during the pregnancy and after birth. In a recent evaluation of parental experiences in the face of a life-limiting prenatal diagnosis, 52% of families reported creating birth plans that focused on comfort-care for the remainder of the pregnancy, delivery, and neonatal care, 27% requested some degree of intervention but placed limitations on more extreme levels of life support after delivery, and 25% requested full interventions and monitoring during the remainder of the pregnancy and life-sustaining therapies after delivery (24). Understanding the value system behind parental-decision making, building a supportive longitudinal care relationship, and guiding goals of care discussions are critical roles of the perinatal palliative care team in creating a comprehensive palliative care plan of care encompassing the remainder of prenatal care, perinatal care including preferences about delivery, and post-natal/neonatal care. The authors use the terms palliative care birth planning throughout the article to describe this processes and palliative care birth plan to describe the document created to communicate the wishes to the care team.

## Parental Decision-Making Experience With a Prenatally Identified Life-Limiting Diagnosis

When a life-limiting diagnosis is made, many variables impact decisions regarding pregnancy continuation and subsequent choices for further care. These factors include maternal age and medical history, religious beliefs, cultural values, severity of anomalies, expected prognosis, gestation at diagnosis, and socioeconomic status (25–28). Additionally, many families consider the worth of their baby, potential quality of life, and personal ethics in the context of the known information (29–31). Several studies have shown the impact of religion on decisions for pregnancy continuation and decisions regarding care after delivery (32, 33). There are times this can lead to feelings of hopefulness, peace, or acceptance. Alternatively parents may experience fatalistic beliefs or fears related to decisions that may be viewed as deviating from their religious beliefs (28). Knowing these factors can help the care team better understand families' decision-making processes as well as help support them through this journey. There are times when personal values and cultural beliefs conflict and families can become lost, experiencing great emotions and turmoil at times leading to ambivalence in the difficult decisions they are asked to make (34).

A qualitative study that described if and how parents decide to proceed with a pregnancy when a diagnosis is made highlighted a three phase process families experience that includes information gathering, assessing implications of multiple uncertainties, and ultimately decision making (35). The manner in which information is presented, as well as parental perception of provider's views, affect the parents' experience and potentially informed decision making (17). During the information gathering phase, providers must be conscious of framing bias and the possibility for this to influence parental choices, as evidenced by the finding that families are more likely to seek interventions if outcomes are portrayed in terms of survival (32). Poor choice architecture and framing bias can be contradictory to the effort to provide non-directive counseling prenatally. Additionally terms such as “lethal,” when used for life-limiting conditions, can convey a sense of finality to families, can leave them feeling hopeless, and may alter the decisions made for the remainder of the pregnancy and afterwards (36). Families have shared experiences of hearing insensitive or rushed remarks that cause lasting trauma (8, 37–39). For example, phrases such as “incompatible with life” were found to be insensitive and unhelpful by parents (40). Families take information in the context of their pre-existing views of disability, parenthood, certainty of the information received, their journey to pregnancy, and their support system when making subsequent decisions (41).

Studies of parental experiences show the importance of clear, concise communication at the time of diagnosis. Simultaneously, acknowledging uncertainty and reviewing further plans for testing or follow up in a timely manner is important (42). Families desire support, empathy, and acknowledgment of the difficult situation they are in and the complexity of decision making (43). Parents want information and support as they continue through their pregnancy journey (44). Often, multidisciplinary support and subspecialty care helps families feel that their questions are being addressed and they are receiving comprehensive care that ultimately aids in coping with the diagnosis (43, 45–47).

Unfortunately, many families feel that this information is poorly communicated. Reports indicate families feel discussions are framed based on providers' view of quality of life which may be vastly different from theirs. Families appreciate providers who offer comprehensive care and planning as well as encouragement to embrace the moments and milestones of their pregnancy (40). Providers must utilize reflexive practices so that members of the treatment team are aware of their own views and how information is portrayed during this vulnerable time (17). Families desire continued support while taking time to process the information and do not wish to feel pressured in to making delicate and important decisions. Parents also want providers to learn about them and their baby as individuals which creates the feeling of significance and that their child matters to the medical team (30). Families also appreciate continuity of care and consistency. Pregnant women and their support people are most comfortable when there is a dedicated provider or care team who takes the time to know them and is willing to explore the fears, questions, and uncertainty that lies ahead with them. Families appreciate learning about all support and treatment options from providers as opposed to seeking resources and information on their own (40, 48).

When the decision is made to continue the pregnancy, the parental experience evolves. Recent literature describes parents' experiences and emotional processing with continuing a pregnancy with a lethal fetal diagnosis (44, 49–53). While variation exists in how individuals cope and process information, an overarching theme is not wanting to have regrets for the remainder of the pregnancy and the decisions made. Parents experience a variety of emotions ranging from shock to existential crisis and ultimately life remodeling as they learn about the diagnosis, live with it, experience the birth and at times the death of their child. Across all of these phases, parents have a variety of coping strategies. As they begin to comprehend the implications of the diagnosis, parents seek information, and use that to revise goals for the pregnancy. Then parents may begin to visualize concrete plans for the remainder of the pregnancy, labor and delivery, the birth, and possibly the death of their child. While parents are preparing, they feel as if they are advocating for their baby, one of the most important responsibilities of parenting. During this time, in addition to the support from family, community, and social networks, families rely on the support of the medical providers (44, 50). Psychosocial support enhances their ability to process the information and be active in truly shared decision-making (51–53). As every individual processes information differently and requires different types of support, it is imperative that providers learn about their patient and listen to them so that the medical team can tailor the care to the unique needs of the family. It is likewise important that providers understand the stages and phases parents go through while processing the diagnosis so that they can recognize where their patient is along the journey and as a result provide the appropriate support and anticipate which conversations families are ready or prepared to engage in.

When a woman learns her fetus has a life-limiting diagnosis, she begins to grieve the loss of a baby she is already attached to (54, 55). Some have noted that receiving the diagnosis meets the criteria of a traumatic experience (17, 56). It is important to recognize that this is true for not just the pregnant woman but her partner, if she has one, and family as well. As a result support should be provided to all parties (57). Healthcare providers need to be equipped to provide psychosocial and bereavement support shortly after the diagnosis is made, through the remainder of the pregnancy and afterwards (58, 59). The support and experience the woman and her family receive has impact far beyond the current pregnancy (60). Initial reactions often include a complex dynamic of grief, guilt, hopelessness, and anger (17, 28, 34, 43, 61). At the same time, parents may maintain a feeling of joy and that their baby is a blessing (62). Families are facing having to make difficult and imperfect decisions that impact this current pregnancy and their relationships afterwards (17, 29, 62).

## Role of Perinatal Palliative Care-Supported Advance Care Planning in Enhancing Opportunities to Parent

The importance of quality, collaborative perinatal palliative care is well-recognized (63–65). When parents choose to continue a pregnancy with a life-limiting diagnosis, they have the unique experience of simultaneously grieving the future for which they have hoped, embracing the milestones of the pregnancy, and planning for how they wish to spend the time they will have with their baby. Many will experience a loss of identity and a sense of isolation (39). Parents often seek the support of their medical providers who have familiarity with what is initially foreign and often overwhelming to them (66). Parents also seek opportunities to feel or regain a sense of control. By being able to make decisions about the remainder of the pregnancy and care of their baby, they are given an opportunity to actively parent and may feel a sense of empowerment (50, 66–68). As the pregnancy progresses, parents continue to bond with their baby. They seek respect for the child's life and shared celebration of the pregnancy milestones and mementos such as maternity pictures, heartbeat recordings, detailed ultrasound images, pregnancy journals, or intentional family activities or rituals. By providing guidance, exploring value systems and goals of care, and creating a space for questions and fears, PnPC teams help families feel supported as parents plan for the life and possibly death of their baby (66, 69, 70).

While discussing the birth and expected death of their child can be difficult for families, the process of creating a palliative care birth plan is also therapeutic and grants families a sense of control and confidence in sound communication of their needs, requests, and hopes (67). Exploration of goals of care, assistance in framing limitations of intervention, and advocacy for sound symptom management are best supported by palliative care trained providers. In the process of advance care planning, PnPC team members balance supportive listening and exploration of family value-systems with facilitation of discussion of goals and the translation of those goals in to medical care plans (10–12, 19, 71–73). The therapeutic relationship that develops between the family and providers leads to increased satisfaction and valued experiences during a difficult time. PnPC providers utilize the process of birth planning as a tool to explore meaningful support from the medical team and ideals the family visions for the remainder of the pregnancy and time with their child. When a family knows that their wishes are heard, it validates their parental role (67, 74). Families find joy in the opportunities to love, parent, and make memories with their baby even before the delivery (75). These experiences are chances to celebrate their child's life and to begin to form an enduring memory and legacy.

The experience of parenting and legacy making are included in the concept of the palliative care birth planning and can occur during the prenatal, perinatal and neonatal care phases. PnPC providers should facilitate exploration of how families seek opportunities to parent, make memories, and create a legacy as the PnPC team is well-equipped to anticipate or suggest what opportunities, choices, and options are available and may be important to the family (40). Photography, recordings, sibling involvement, creation of tangible keepsakes, bathing, and family-centered or faith-based rituals are commonly valued memory making opportunities during the remainder of the pregnancy and afterwards (76). Some families also seek to find meaning beyond their interaction with their babies and hope to create a lasting legacy for their child through organ/tissue donation, contributions to research or education, or breast milk donation. As advocates for family well-being, PnPC providers often balance planning for these valuable memory-making and life-honoring opportunities with careful watch for grief and complicated bereavement. This is especially true when carefully planned time with a baby after delivery or the opportunity to donate organs after death does not come to fruition as hoped.

## Exploring Goals of Care During Perinatal Palliative Care Consultation

The perinatal palliative care birth planning process is ideally longitudinal and interdisciplinary. Families benefit from multiple points of contact with care teams. Many PnPC providers advance conversations incrementally through several visits, focusing initially on rapport building and ensuring appropriate medical understanding and prognostic awareness before transitioning to exploration of goals of care and birth planning (10). This allows for manageable amounts of information to be received by families and gives space for reflection and formation of questions. PnPC is interdisciplinary, utilizing care team members with specific training in psychosocial assessment and spirituality as well as communication and medical expertise to optimize a climate of shared decision-making (77). Parents appreciate receiving balanced information about the diagnosis and all treatment options before making decisions. They appreciate being part of the care discussions, having their voices heard, acknowledgment of their difficult situation, and ongoing support (30, 71). They rely on providers to encourage them to express their hopes, fears, and goals (78). Providers and parents collaborate to make decisions and plans, balancing autonomy and parental authority with medical recommendations and prognosis (79).

Discussing goals of care is a key communication skill when caring for patients with a complex or life-limiting illness (69). These conversations generally begin with exploration of parental values, goals, and fears leading to creation of a care plan that honors these views and wishes. When exploring goals of care with the family, it is pertinent providers assess the family's understanding of the diagnosis, prognosis, and range of treatment options. If providers can be present and supportive during these emotional conversations, there is an opportunity to learn more about the family, their views of quality of life, and how they make difficult decisions. This allows the provider to better understand and align with the family and ultimately helps to develop a care plan that is consistent with the family's goals and values. Early discussion of goals of care allows for time to reflect and plan compassionately and thoughtfully (80). For example, wishes for fetal monitoring during labor or newborn medical intervention should be established before families present for the birth of their child (7, 81–83). It is important to acknowledge that goals of care may evolve throughout the course of a pregnancy or after delivery as information available or the family's views and experience may change. For example, if an infant is unable to ventilate despite artificial respiratory support, this could be an opportunity to pause and reevaluate goals of care in the context of the evolving clinical situation. Ongoing perinatal palliative care support provides families with a safe place to explore the uncertainty of life-limiting fetal diagnoses and a team committed to careful communication of goals of care and palliative care birth plans (5, 67). Table 2 uses a modified REMAP framework to outline key components of exploring goals of care in the setting of life-limiting fetal diagnosis (79).

**Table 2:** Exploring goals of care for families facing life-limiting fetal diagnoses [modified REMAP framework (80)].

**Reframe medical understanding and prognostic awareness** • Help family explore “What does all of this mean?” • Acknowledge uncertainly and balance of “hope and reality” **Expect emotion** • Ask open ended questions (i.e., what are you most worried about?) • Validate and respond to emotion. Be okay with silence **Map out value system family uses to makes decisions** • Inquire about faith, spirituality, religion • Help to put those values in context of child's care (i.e., what is most important to you when thinking about how you want the team to care for your child?) **Align with values and hopes** • Reflect and summarize what we are hearing • Seek clarification and verification **Propose a plan** • Suggest how identified goals may be achievable • “I am hearing you ask that we create a plan that focuses on _______ for Baby's care after delivery” (time, comfort, better understanding diagnosis, etc.)

## The Palliative Care Birth Plan as an Advance Care Plan

While the process of palliative care birth planning goes far beyond the tangible specifics of care surrounding delivery, the palliative care birth plan itself is important for families and providers. The palliative care birth plan functions as a communication and advocacy tool among care team providers. Providers caring for the mother often have different perceptions of family's understanding than providers caring for the baby (67). In order to fully support the family and provide seamless care, there must be open communication and shared understanding between all team members. The care team for a family delivering an infant with potentially life-limiting diagnosis will be comprised of both obstetrical and neonatal providers, including physicians, advanced care nurse practitioners, nurses, respiratory therapists, and more. The birth plan reflects the exploration of goals of care, summarizes the pregnancy journey, and shares the voice of the family while simultaneously documenting requests to all potential medical care providers for care during labor, delivery and the neonatal period for both mother and baby.

In order to meet the needs of both the families and providers, the palliative care birth plan should be viewed as an advance care planning document. Advance care planning includes deciding what medical care is desired in the setting of a life-limiting illness, exploring and sharing personal values, and documenting and communicating wishes (84). Each palliative care birth plan is unique to the family that creates it and serves as a tool to share their wishes and medical decisions regarding care of the mother and baby for the remainder of the pregnancy, around the time of delivery, and the subsequent neonatal care. As palliative care birth plans typically reflect discussions with medical care teams, the documents are generally created with the direct support of perinatal palliative care providers.

Palliative care birth plans include a breadth of information pertinent to providers involved in the care of the mother and baby. While many templates exist, key information should be included that will aid in seamless care during the delivery process and afterwards. General information such as the parent's names, the baby's given name (as many EMR's utilize automatic naming conventions based on the mother's name), and the parents' understanding of the diagnosis is important. Likewise, contact information for support persons should be readily available. This allows the care team to delegate tasks such as contacting family to others so that the parents can focus on the labor and delivery process while still having access to the support they desire. The document can include some specifics about goals during the pregnancy including celebrating milestones, memory-making such as pregnancy photography and preferences for follow up and delivery location. Wishes for maternal care during labor and delivery should be addressed ahead of time in the palliative care birth plan. This includes if the mother desires fetal monitoring, if she would want a cesarean delivery, analgesia, who should be present during the time of labor, and any special requests. Some women request music, aromatherapy, dim lights, or time alone with their partner during labor as a way to relax during this potentially stressful, exciting, and anxiety-provoking time. If they baby is live born, the medical care of the baby including specific requests for resuscitation or interventions should be clearly documented. This is an opportunity to express to the care team the wishes based on detailed discussions that have occurred throughout the pregnancy. Common discussions addressed include options for comfort measures only, non-invasive medical interventions, and invasive medical interventions. Beyond those goals, parents may have specific requests for feeds, testing, and symptom management. Equally important, if applicable, are wishes for end-of-life care including location of death, symptom management, organ donation, autopsy, and funeral arrangements. One of the most important components of the birth plan to parents is the opportunity for memory-making. Who is included and what keepsakes or rituals are important is unique to each family. Table 3 outlines details important to a birth plan.

**Table 3:** Key components of a palliative care birth plan.

**Important information for the care team** • The parents' names • The baby's name (if known) • A diagnosis for the baby (if known) and a summary of what the family has been told to expect around/after the time of delivery. Any pertinent medical information should be included • Names and numbers for important members of the care team (obstetrician, pediatrician, geneticist, other subspecialists) • Names and numbers for important support persons (family, clergy, friends who should be involved) **Wishes for labor and delivery** • Vaginal or cesarean birth ∘ Indications for when they would want a cesarean delivery • Fetal heart monitoring during labor • Analgesia for mother • Special requests during labor • Who should be present • Who should cut the umbilical cord • Preferences for if the baby is stillborn **Wishes for medical care of the baby** • Invasive interventions vs. comfort measures only ∘ Any specific interventions they would or would not want • Wishes for holding the baby • Wishes for delaying routine procedures or providing them while the baby is in the parent's arms • Wishes for feeding the baby • Wishes for medications • Wishes for additional testing **Wishes for memory-making and support** • Who should be in the room after the baby is born • If there are siblings/family members, who will talk with them and how do they wish to be involved • Photographs and videos • Keepsakes: footprints, handprints, locks of hair, crib card, ID bands, blankets, clothing, heart beat recording • Wishes for bathing the baby and special outfits • Spiritual rituals/wishes **Plans for if the baby survives the first day** • If the family would like to take their baby home vs. receive end of life care in the hospital • If they wish to be discharged home ∘ Code status at discharge ∘ The name of the hospice/home care that will support them ∘ Anticipated care needs at home ∘ Information about whom to notify if baby dies at home **Plans for if the baby dies before discharge** • Plans to ensure the baby is comfortable during the dying process • Wishes to keep the body in their room • Wishes for organ/tissue donation if eligible • Wishes for autopsy or further testing • Funeral home information • Special wishes about transportation of the body **Any other additional requests the family may have**

Families identify palliative care birth planning as extremely important in preparing for the medical management of their child (67). It is not uncommon for families to request supportive care, varying degrees of resuscitation/delivery room interventions, and even life-sustaining or life-extending therapies for their child with a life-limiting fetal diagnosis. In creating requests for care, parents balance desires with fears. Common hopes and desires include meeting the child alive, enjoying time together as a family, and even providing a life and home for the child. These are balanced and contrasted with fears of pain, suffering, and extreme medical complexity (24, 79). The PnPC providers consistently utilize goals of care discussions and multidisciplinary birth planning to support families and address this duality resulting in requests for perinatal and post-natal care that is consistent with family values and rooted in medical understanding and prognostic awareness (85).

As much as the palliative care birth plan is a voice for families and communicates their advance care plan to the team, maternal and neonatal medical teams often seek expanded details on the medical care to be provided (67). It is important that all providers understand conversations that having taken place about the diagnosis, prognosis, and treatment options available. This often requires thoughtful and detailed documentation beyond the palliative care birth plan in the medical record. Delivery hospital teams should be directed toward details of collecting post-natal genetic samples or providing further neonatal assessments. Furthermore, documenting a family's decision-making structure and unique needs helps providers to partner with them during labor and delivery and provide optimal family-centered support while minimizing distress and miscommunication. An outline of pertinent information to include in provider notes around advance care planning in the form of birth planning can be found in Table 4.

**Table 4:** Important components of provider notes on birth planning.

**Perinatal palliative care meeting note** Team members present: Timing and reason for the visit Family members present at the meeting The name of the baby Maternal history Pertinent OB labs Results of fetal testing and imaging Name of the subspecialists involved in the medical care Social History Share with others the important insight you have gained. Talk about the family structure, siblings, jobs, support structures, and faith. It is also helpful to talk about additional stressors and how the individuals involved in the shared decision-making are processing and coping with the diagnosis Impressions, Counseling, and Plan Description of anomalies Counseling on prognosis and potential complications Who was present and a detailed description of what the family has been told to expect as far as morbidity, mortality, and anticipated medical challenges (including the ranges and both short and long term complications). Discuss what treatment options, care paths, and interventions have been offered and how that is expected to change the disease trajectory. Goals of Care Discuss the wishes of the family that they have expresses (comfort measures, non-invasive interventions, invasive interventions). Explain the various options and interventions discussed in detail (this is a guide for the providers). Also expand on any uncertainty and things that are important to the family if their goals are conflicting or cannot be met. Discuss labor and delivery wishes in the delivery room, code status, site of newborn care, anticipated procedures, or medications (help the providers who haven't met the family prepare for what to anticipate/expect), memory-making, religious ceremonies, plans for if the baby dies before discharge, and plans for if the baby survives to discharge

Multidisciplinary fetal care teams have varying formats for discussion and planning upcoming deliveries of complex neonates and babies with life-limiting diagnoses. Discussions including key stakeholders (OB, neonatology, palliative care, etc.) ensure that providers are in agreement and all questions are addressed. Including families, psychosocial and spiritual support staff, and nursing teams may offer the opportunity to ensure interdisciplinary communication, allowing insight in to what is most important to the family and how to collaborate in family-centered-care after delivery. Additionally, palliative care birth plans should be readily available to providers and ideally documented in the electronic medical record before the time of delivery.

## Discussion

When a pregnancy is complicated by a life-limiting fetal diagnosis, the remainder of the pregnancy is vastly different from what was hoped for and expected. The grieving process may start well before the family even meets the baby. Often, families seek opportunities to celebrate and honor the life of their child while simultaneously planning for the ongoing pregnancy and navigating decisions surrounding care of the newborn with a life-limiting condition.

Exploration of goals of care, assistance in framing limitations of intervention, and advocacy for sound symptom management are well-supported by palliative care-trained providers. Multidisciplinary fetal care centers are growing to include perinatal palliative care specialists, who support families in processes of exploring value-systems, establishing goals and requests for medical care, and communicate those care plans with the broader multidisciplinary delivery team. Ultimately, perinatal palliative care birth planning and the creation of a palliative care birth plan with the guidance of an interdisciplinary perinatal palliative care team functions as an advance care planning tool for both families and providers. Further research dedicated to perinatal palliative care practices surrounding birth planning and multidisciplinary meetings as well as family experiences related to advance care planning during complicated pregnancies will lead to improved supportive care of families experiencing life-limiting fetal diagnoses.

## Author Contributions

DC, KE, and AS conceptualized and designed the report, drafted the initial manuscript, and approved the final manuscript as submitted.

## Conflict of Interest

The authors declare that the research was conducted in the absence of any commercial or financial relationships that could be construed as a potential conflict of interest.

## Abbreviations

PnPC: Perinatal Palliative Care.
